# Identifying critical recruitment bottlenecks limiting seedling establishment in a degraded seagrass ecosystem

**DOI:** 10.1038/s41598-017-13833-y

**Published:** 2017-11-01

**Authors:** John Statton, Leonardo R. Montoya, Robert J. Orth, Kingsley W. Dixon, Gary A. Kendrick

**Affiliations:** 1 University of Western Australia, Oceans Institute, Perth, 6009 Western Australia Australia; 2 Virginia Institute of Marine Science, College of William and Mary, Gloucester Pt., 23061 VA USA; 3 Department of Environment and Agriculture, Curtin University, Bentley, 6102 Perth, Western Australia

## Abstract

Identifying early life-stage transitions limiting seagrass recruitment could improve our ability to target demographic processes most responsive to management. Here we determine the magnitude of life-stage transitions along gradients in physical disturbance limiting seedling establishment for the marine angiosperm, *Posidonia australis*. Transition matrix models and sensitivity analyses were used to identify which transitions were critical for successful seedling establishment during the first year of seed recruitment and projection models were used to predict the most appropriate environments and seeding densities. Total survival probability of seedlings was low (0.001), however, transition probabilities between life-stages differed across the environmental gradients; seedling recruitment was affected by grazing and bioturbation prevailing during the first life-stage transition (1 month), and 4–6 months later during the third life-stage transition when establishing seedlings are physically removed by winter storms. Models projecting population growth from different starting seed densities showed that seeds could replace other more labour intensive and costly methods, such as transplanting adult shoots, if disturbances are moderated sufficiently and if large numbers of seed can be collected in sufficient quantity and delivered to restoration sites efficiently. These outcomes suggest that by improving management of early demographic processes, we could increase recruitment in restoration programs.

## Introduction

Restoration of terrestrial vegetation has moved from transplanting or seeding target plant species to restoration of biodiverse plant communities, building demographic resilience and replacing ecological function^1^. This is not the case for restoration of the marine vascular plants, the seagrasses^2^. Recent reviews show that most seagrass restoration programs are little more than pilot-scale transplantation exercises where the resilience and recovery of the transplants are only followed for a short period and are mostly unsuccessful^2–6^. In addition, these restoration efforts come at a high cost, from $US33 000 ha^−1^ to more than $US3.3 million ha^−1^ depending on the project, species being transplanted and geographic region^4^. More importantly, the scale of transplanting (<10’s of ha; e.g.^7^) cannot hope to alleviate the massive scale of restoration compounded by the increasing rate of seagrass loss (100’s km^2^ per decade^8^). While seeding has recently emerged as an effective tool to restore large areas of degraded seagrass habitat for one species, *Zostera marina* in the USA (100’s–1000’s ha^9^), and at a reasonable cost (annual re-seeding $US10 000 ha^−1^ R. J. Orth Pers. Comm), the science behind seed-based approaches is largely underdeveloped for the majority of seagrass species and severely lags behind their terrestrial counterparts. Clearly it is time to move from this situation to applying terrestrial plant models of seed-based restoration to seagrasses.

Life-cycle population models have been valuable in the development of terrestrial restoration theory and practice. In particular these models allow for the quantification of early life-stage transitions from seed, to germinant, to emerged seedling, identifying which of these transitions are the most limiting in plant recruitment^10^. Armed with this knowledge restoration practitioners can target those life-stage transitions most responsive to management^10,11^. However, only recently have seagrass ecologists investigated the importance of early life-history elements for seed-based recruitment in seagrasses, challenging the paradigm that seed recruitment is of little consequence to the persistence, maintenance and recovery of seagrasses^12–14^. As a result, early life-history demographic studies are remarkably few among seagrass species. This is a crucial research gap given that our ability to restore and manage seagrass populations effectively is limited since we have little understanding of the demography of early life-history components.

Determining the magnitude of life-stage transitions limiting seedling establishment is the first step in understanding the demography of a plant species and the influence of the local environment on successful recruitment. Previous research on recruitment from seeds in seagrasses have found high mortalities between seed settlement and seedling establishment, where poor survival of seedlings was driven by physical disturbance associated with winter storms^15^. Similarly^16^, observed that losses of *Zostera marina* seedlings during winter in north-eastern USA were not related to poor germination rates (internal) but rather to physical forces (external) such as waves and currents disturbing sediments and removing seeds. Predation and herbivory also influence seedling establishment and their persistence. Seed predation can remove seeds from the recruitment pool^17–19^ and seedling defoliation from herbivory^15^ can reduce the photosynthetic area and depress plant growth and resilience.

**Figure 1:**
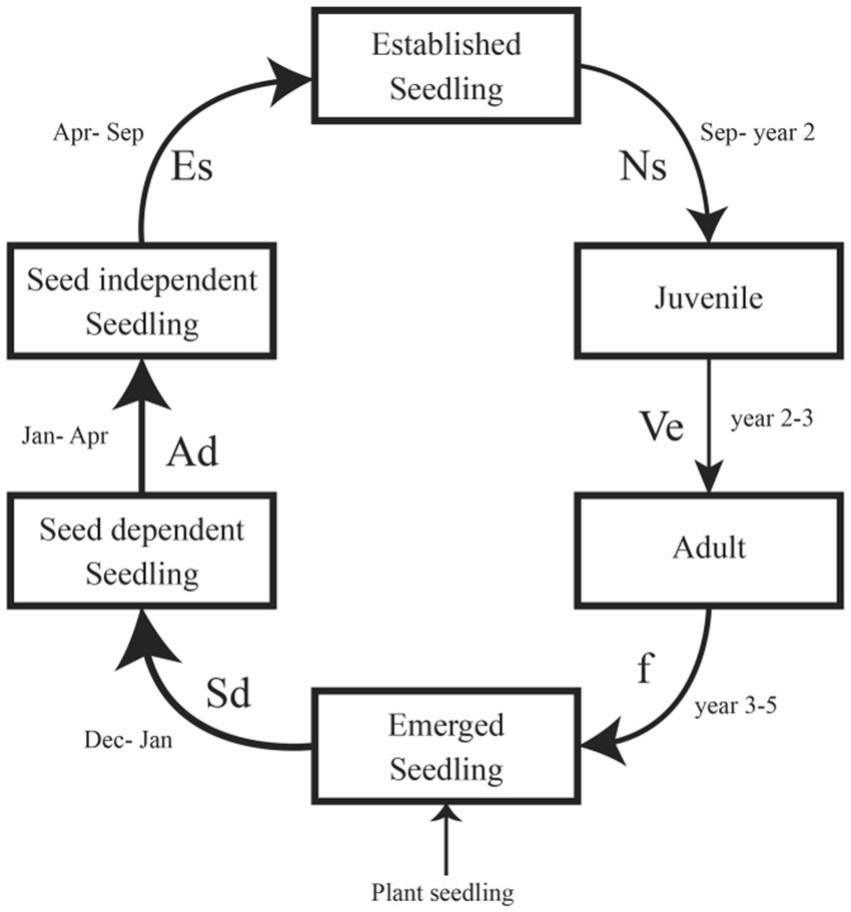
A life-cycle model describing the demographic stages (boxes) and transitions (arrows) *Posidonia australis* follows to adulthood and the months each transition spanned. The transitions included; Seed-dependency, when seedlings are highly dependent on seed reserves, Sd (December_yr1_–January_yr1_). Seedlings then undergo an extended period where they continue to draw nourishment from maternally-derived reserves but there is greater uptake and assimilation of resources from the environment due to production of photosynthetically active leaves and development of a small but functional root system; autonomous development, Ad (January_yr1_–April_yr1_). By the end of this period seedlings have exhausted the majority (~90%) of their seed reserves and are relatively independent of their seed. Seedlings then become fully integrated into their environment upon exhaustion of the seed reserves; seedling establishment, Es (April_yr1_–September_yr1_). Production of new shoots, Ns (September_yr1_–year 2) typically occurs in the months following seedling establishment and seedlings become Juveniles. Juveniles transition into adults after plants undergo horizontal vegetative expansion, Ve (year 2–3). Adults typically become reproductively mature, f (fecundity), between years 3–5.

For seed-based restoration programs utilizing one of the most widely distributed genera in Australia, *Posidonia*, initial population size is limited by a series of transitions from seeds to seedlings that initially are dependent on seed reserves, to seedlings independent of seed reserves then juveniles (>1 year old) to reproductive adults (Fig. 1). Although recruitment of a seed into the adult population depends on these early life-stage transitions^20^, few seagrass demographic models acknowledge these early life-history components (but see^16^). Instead current practices have quantified seed recruitment as the proportion of seeds that establish as seedlings, combining all the transitions a species undergoes into one (e.g.^14^). Without an understanding of the proportion of seeds transitioning through early life-stages leading up to seedling establishment, we cannot identify and manage the processes driving population dynamics (and recruitment failure) in restoration programs^21^.

The genus *Posidonia* produces viviparous seeds that are released, settle and colonise sediments over a 2–3 month period in both the Northern (e.g. *Posidonia oceanica* ^22^), and Southern (e.g. *Posidonia* spp.^23^) hemispheres. Without the capacity to store seeds for later restoration as utilized in *Zostera* seed-based restoration^24^, *Posidonia* seeds need to be deployed immediately or grown *ex situ* in culture to transplantable seedlings (e.g. *Posidonia australis* ^25,26^). Previous seed-based restoration experiments have not been successful for *Posidonia* but the scale of seeding was limited to 100’s of seeds, the transplanting environment was inadequate and with little characterization of the physical disturbance regimes, and monitoring of recruitment success limited^2,6^. Here, we address these limitations by investigating the demographic consequences of seed-based restoration and the effect of seed density on survival for *Posidonia australis* in temperate Western Australia.

The specific objectives of this study were to determine through a large field-based seed restoration experiment: the mortality of early life-history transitions during establishment of seagrass seedlings within historically degraded sites, and; how they vary spatially along gradients in disturbance resulting from water motion, grazing and bioturbation. We then use a transition matrix model utilizing the outcomes of the experiment to investigate what life-stage transitions most limit seedling recruitment and population growth in the first year of seed recruitment, and; to make predictions from the matrix model as to the most appropriate environments and seeding densities required for seed-based restoration in the seagrass, *Posidonia australis*.

## Results

### Restoration locations and environmental characteristics

**Figure 2:**
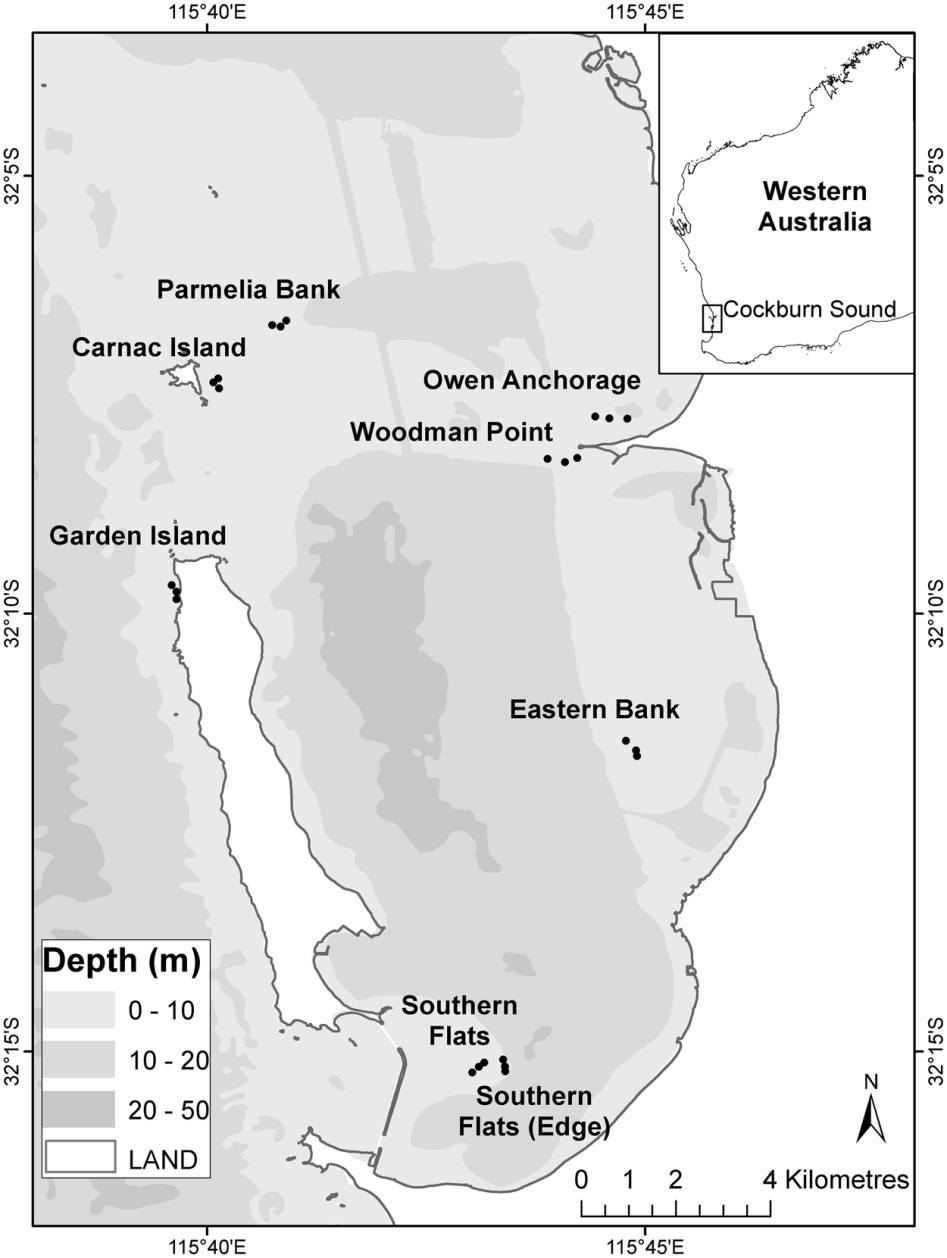
Study area with position of all Locations and their Sites within bare sand in Cockburn Sound (Inset: Cockburn Sound, Western Australia). Figure created in ArcMap (ESRI 2011. ArcGIS Desktop: Release 10. Redlands, CA: Environmental Systems Research Institute).

The locations varied in depth from ~2.4 m, recorded at the southern end (Southern Flats, SF) of the study area, to ~8.4 m (Parmelia Bank, PB), north of Cockburn Sound (Table S1, Fig. 2). There was a strong wave-exposure gradient, which was greatest in the west and north and least in the east and south. The shallow sites varied in exposure to wave action from protected in the south (SF), to exposed, in north-western locations, Carnac Island (CI) and Garden Island (GI). Owen Anchorage (OA) and Woodman Point (WP) had depths of ~4.5 m and were located close to the mainland at the northern end of the study area but had moderate exposure to waves. Deep locations also had contrasting exposure, the most exposed being Parmelia Bank (PB) and the least exposed was the eastern bank (~7.5 m) in Cockburn Sound (CS).

The sediment distribution across locations was normal, with mean grain size of medium sands (~0.3 mm) (Table S1). GI, CI and PB sediments were well-sorted while the southern and eastern locations were moderately sorted. Sites located at the southern end (SF) contained large pieces of biogenic material such as bivalves shells. The size distribution was symmetrical on all the sites but slightly skewed towards the coarse at WP. Organic matter content on surface sediments was ~2.7% (2.3–3.0%) of sediment dry weight, with some presence of seagrass matte (Table S1). The light availability on the sea-floor was simultaneously recorded at SF (shallow), OA (mid-depth) and PB (deep) (Table S1). SF recorded the highest daily light availability of ~10.6 mols photons m^−2^ d^−1^ (±1.96 mols photons m^−2^ d^−1^). There was a 70% decrease in light availability from shallow to deep, with a maximum of 3.28 mols photons m^−2^ d^−1^ recorded at PB (±0.91 mols photons m^−2^ d^−1^). Given that *Posidonia* seagrasses occupy this depth suggests that light availability is within a suitable range for this species across our study area.

During winter, when wave conditions were greater due to storm fronts moving from north west to south west, maximum significant wave height (Hs_max_) recorded at the most exposed north-westerly site (PB) was 0.9 m (Table 1), with long periods from 8–25 s. Hs_max_ decreased by 45% at sites with medium exposure and 78% at the most sheltered site in southern Cockburn Sound (SF) (Table 1). In total ~6 large storm events were recorded over the two months of data collection during winter 2014. Storm events were defined as daily averaged Hs_max_ surpassing the 75^th^ percentile. The longevity of these events varied from short extreme events to systems lasting up to ~3–4 days (Table 1).

**Table 1:** Environmental wave conditions (significant wave height, Hs) from spectral analysis of the frequencies 8–25 s during Winter (Aug–Sep 2014).

Location	Depth (m)	Hs (m)	Hs_MAX_ (m)	Hs 75^th^ percentile (m)	Days > 75^th^ percentile	Events > 75^th^ Percentile
SF	2.41	0.08	0.21	0.10	15	8
CS	7.48	0.14	0.28	0.17	14	6
WP	4.29	0.24	0.50	0.29	12	5
OA	4.55	0.19	0.47	0.23	13	6
CI	2.95	0.31	0.54	0.37	13	5
PB	8.43	0.46	0.92	0.56	13	6

Southern Flats (SF), Cockburn Sound east bank (CS), Woodman Point (WP), Owen Anchorage (OA), Carnac Island (CI), Parmelia Bank (PB).

In comparison to previous year’s storminess, our seeding year was a relatively harsh year with several storms arriving from the north, particularly in the month of July (Fig. S1). In 2010 and 2013, despite several storms originating in the southwest, these were considered relatively benign years in terms of storm activity arriving from the north (Fig. S1).

**Figure 3:**
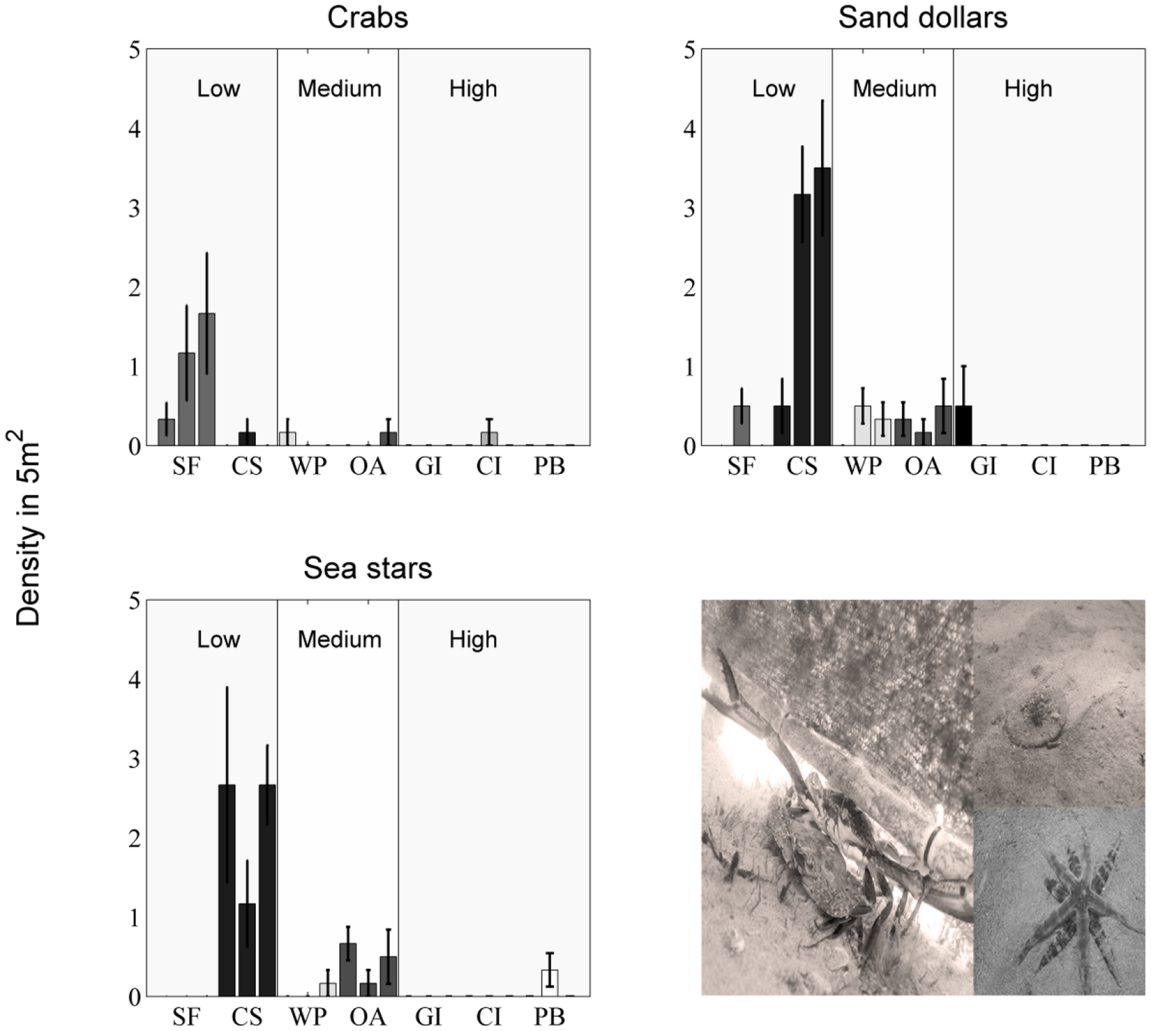
Faunal densities observed with 5 m^2^ (5 m × 1 m transect) at locations with low, medium and high wave-exposure. Columns are means (±1SE). Image on bottom right shows (clockwise); Blue swimmer crabs (male [top] and female [bottom]), sand-dollar and sand sea-star.

Benthic bioturbators and potential seed predators were more abundant in sheltered inshore sites than offshore wave-exposed sites. Blue-swimmer crabs (*Portunus armatus*), sand-dollars (*Peronella lesueuri*) and sand sea-stars (*Archaster angulates*, Fig. 3) were the most abundant benthic fauna at sheltered CS, SF and SFe, with densities of 1–4 individuals per 5 m^−2^ transect (Fig. 3). At moderate wave-exposed sites (WP & OA) there were lower densities of the bioturbators/grazers (<1 individuals per 5 m^−2^ transect), and few individuals were observed at offshore locations (GI, CI and PB).

### Life-stage transition probabilities at restoration locations

**Figure 4:**
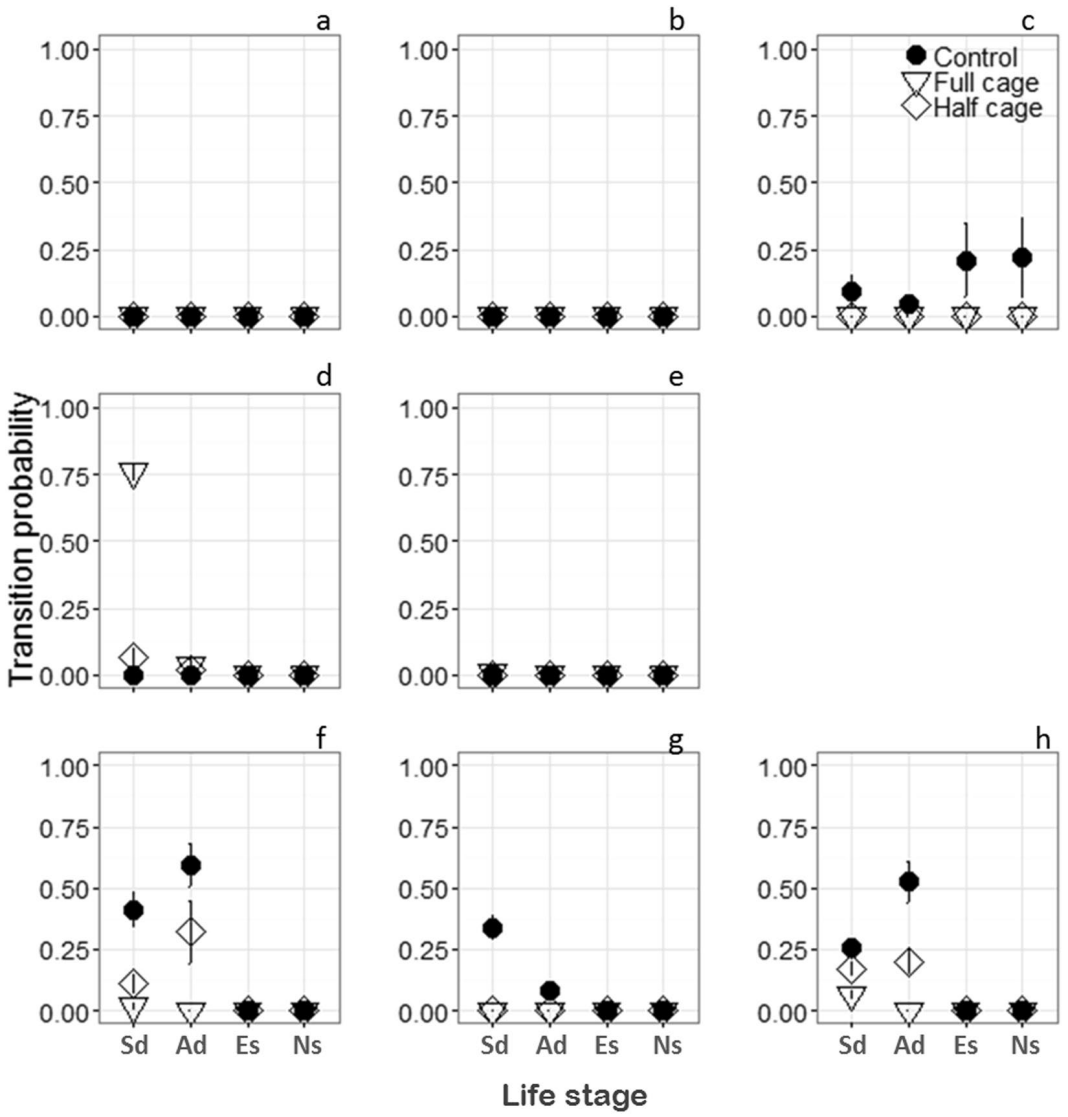
Life stage transition probabilities at the different Locations within Cockburn Sound; Southern Flats (**a**), Southern Flats edge (**b**), Cockburn Sound eastern bank (**c**), Owen Anchorage (**d**), Woodman Point (**e**), Carnac Island (**f**), Garden Island (**g**) and Parmelia Bank (**h**). Life-stage transitions - seed-dependency (Sd), autonomous development (Ad), seedling establishment (Es), survival to juveniles (Ns). Fauna exclusion cages were tested at all sites and included; full cage enclosures, half-cage enclosures (procedural control) and uncaged controls.

Across all locations the total survival probability of *P. australis* seedlings was low (0.001) with total survivorship driven by one location with low wave exposure and in deep water (CS, Fig. 4). Across low and moderately wave-exposed, shallow locations (SF, SFe, OA, and WP) seed-dependency (1 month, the life-stage where seedling growth is 100% dependent on seed reserves) was the transition with the lowest (100% loss) survival probability (p < 0.001, Fig. 4). In wave-exposed locations (CI, GI, PB) seedling survival was high during seed-dependency with transition probabilities of 0.25–0.41. The transition probability to seed-independent seedling was also high for CI and PB (0.50–0.60) but lower for GI (0.15), yet were not significantly different (p = 0.34). However, in wave-exposed locations during the seedling establishment life-stage, we recorded 100% losses of seedlings, resulting in no seedlings surviving past this life-stage transition for exposed locations.

### Limiting life-stage transitions at restoration locations

The use of transition matrices allowed us to assess the importance of the different life stages, and their effect on the stability of the population (λ). However, we are excluding clonal expansion from this analysis. This exclusion was purposely made to capture the effect of a population establishing specifically from seeds. For all the seedlings as a single population, we obtained a λ smaller than one (mean = 0.49, range = 0.2–0.7), meaning a decaying population. We obtained the greatest λ for the sheltered deep sites (λ = 0.71). The exposed scenarios on the other hand gave a λ of only 0.21, reflecting the general decline of the population across all the stages, with complete mortality during the establishment stage (Table 2).

**Table 2:** population growth rate (λ), sensitivity (S) and elasticities (E) analyses for each life stage transition.

	λ	Sensitivities & Elasticities						
			Sd	Ad	Es	Ns	Ve	f
All sites as a single population	**0.49**	*S=*	***0.62***	0.23	***2.17***	0.09	0.30	0.01
		*E=*	0.18	0.18	0.18	0.18	0.12	0.18
Low exposure; Shallow (SF, SFe)	0.21	*S=*	***8.24***	*2.29*	0.00	0.00	0.91	0.00
		*E=*	0.02	0.02	0.02	0.02	0.89	0.02
Low exposure; Deep (CS)	**0.71**	*S=*	***1.37***	*0.68*	0.15	0.13	0.26	0.02
		*E=*	0.19	0.19	0.19	0.19	0.07	0.19
Moderate exposure and depth (WP & OA)	0.21	*S=*	***4.12***	***4.58***	0.00	0.00	0.91	0.00
		*E=*	0.02	0.02	0.02	0.02	0.89	0.02
High exposure; Shallow (GI,CI)	0.21	*S=*	0.01	0.01	**538.22**	0.00	*0.91*	0.00
		*E=*	0.02	0.02	0.02	0.02	0.89	0.02
High exposure; Deep (PB)	0.21	*S=*	0.02	0.01	**581.74**	0.00	*0.91*	0.00
		*E=*	0.02	0.02	0.02	0.02	0.89	0.02

Note that the elasticity during the seedling stage is greater than the vegetative stage for two of the shallow scenarios (in bold). The sensitivities show the specific stages that have greater effect over the establishment (*italic*). Life stages: seed-dependency, Sd (December_yr1_ –January_yr1_); autonomous development, Ad (January_yr1_–April_yr1_; seedling establishment, Es (April_yr1_–September_yr1_); juveniles (new shoot production after Es), Ns (September_yr1_–year 2); horizontal vegetative expansion, Ve (year 2–3). Adults typically become reproductively mature, f (fecundity), between years 3–5.

Sensitivity analysis showed for sheltered and deep sites, population growth was driven by early seedling life-stage transitions (Sd and Ad), while for exposed sites greater sensitivities occur during seedling establishment (Es) (Table 2). The sheltered and deep locations produced greater elasticity values during early seedling development (Sd-Ns) compared to adult life-stage transition (Ve) suggesting an increase in one of these survival transitions would produce a greater change in λ and consequently in all the population (Table 2). The exposed populations both east and west showed the opposite pattern on elasticities; for these populations there was a greater value on the elasticity on the adult stage (Ve = 0.89) than on the seedling transitions (Sd-Ns = 0.02). This suggests that increasing a single survival stage of seedlings would result in little change in overall recruitment success.

### Modelling seeding densities at restoration locations

**Figure 5:**
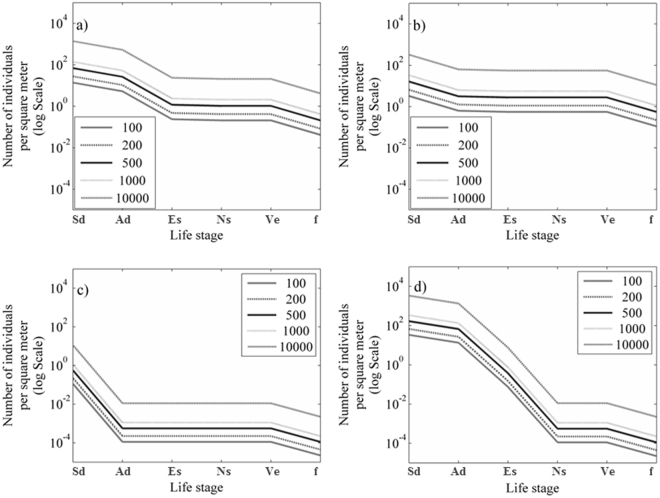
Modelled projection of survivors throughout the transition stages with an increase on the number of seedlings planted. (**a**) All sites as a single population. (**b**) Individuals at the low exposure sites, including shallow and deep. (**c**) Individuals from the mid-exposure sites and (**d**) Individuals at the exposed sites (shallow and deep). Life-stage transitions - seed-dependency (Sd), autonomous development (Ad), seedling establishment (Es), survival to juveniles (Ns), adults (Ve), fecundity (f).

The transitions in survival of seedlings across all stages were evaluated with a hypothetical increase in the number of seeds planted (Fig. 5). Two to 40-fold increases in seeding rates (m^−2^) across sheltered sites increased the number of seedlings surviving to adulthood (Fig. 5a,b) with a proportional increase in survivorship up to 10 individuals m^−2^. In exposed locations the onset of winter storms resulted in complete mortality at any seeding density.

## Discussion

Major recruitment bottlenecks to successful seedling establishment occurred in P*osidonia australis* during the experiment and the drivers of mortality varied at different life history transitions. In the seagrass *Posidonia australis*, successful establishment from seeds was strongly influenced at early life-stage transitions, by gradients in seed predation and dislodgement by bioturbators in summer and storms during winter. A similar approach was used by^10^, who found that seed ‘emergence’ was a major bottleneck in recruitment in arid grasslands. This study has identified that the early recruitment environment has important implications for successful *P. australis* establishment and for identifying what ecological processes need to be managed to increase seedling recruitment in seed restoration programs.

Seeds in the benthic sedimentary environment are in a dynamic state^27^ and therefore an understanding of seed movement^13,28^ and seed fates^14^ is essential for seagrass restoration and management efforts. Secondary dispersal agents (i.e. secondary dispersal is the dispersal after release from parent to sediment surface or after seeding interventions^27^ such as fauna and water movement), will likely drive the patterning of seedling establishment and population dynamics across a marine landscape^29^. Interactions with marine fauna can occur within days to weeks after a seed settling event. For example, in Rottnest Island, Perth, at least six different crustacean taxa predated on *P. australis* seeds within a day of seed release^19^. In Odense Fjord, Denmark, lugworms (*Arenicola marina*) buried 95% of seeds and 75% of seedlings of *Z. marina* below their critical depth within 4–8 weeks^30^. Despite the massive seed losses within the first month of our study, we know little about how the most abundant benthic fauna; Blue-swimmer crabs (*Portunus armatus*), sand-dollars (*Peronella lesueuri*) and sand sea-stars (*Archaster angulates*, Fig. 4) influence *Posidonia* seeds, and whether seeds were completely removed from the recruitment pool or just displaced. Based on the patterning of faunal tracks and pits we observed within our study locations we assume that seeds were dragged or pushed outside and away from our plots by sand-dollars and sea stars, or dislodged, buried or predated by crabs. Clearly, a better understanding of the mechanisms of faunal disturbance along with the movement dynamics and behavioural ecology of these benthic fauna will improve our ability to predict successful recruitment and/or develop strategies to overcome recruitment bottlenecks.

Hydrodynamic forces also have the potential to significantly influence the practice and outcomes of seagrass restoration and conservation by altering the biophysical setting in which a seed recruits. Under extreme storm events the spatial distribution of entire annual seagrass populations can be shifted^31,32^. In Cockburn Sound the water movement during summer tends to be driven by low tidal ranges and local wind circulation from the east and south-west such that most locations are relatively protected by land features (e.g. Garden Island attenuates south-westerly wind-driven seas and oceanic swells). Whereas in winter, locations near the northern boundary of Cockburn Sound tend to be wave-swept, particularly from storm events arriving from the west and north^33,34^. An analysis of hydrodynamic conditions during winter (2014) revealed that at least six major storms crossed the coast, with four such events arriving from the north. Subsequently, in moderate to high wave-exposed locations, we observed high seedling survival prior to the arrival of winter storms, but complete loss during winter.

Repeat seeding over successive years may be the most appropriate strategy to overcome recruitment bottlenecks and enhance *Posidonia* seagrass establishment. Despite our results suggesting that seedlings are less suited to locations with high faunal abundance and high wave-exposed environments, the natural existence of adult stands and small patches of *P. australis* within these environments suggests recruitment has occurred in the past (e.g.^35^) though potentially these events may be highly episodic. In addition, high genetic variation within populations of *P. australis* and high genetic connectivity across the northern section of our study area^36^ infers contemporary recruitment occurs. In this study, determining demographic rates across environmental gradients showed that successful recruitment required benign conditions. For *P. australis*, a species that is an effective seed disperser, high fecundity and no dormant seed bank^13^ could be interpreted as adaptations to the highly variable and disturbed environment they live. ‘Windows of opportunity’ for recruitment limiting seedling recruitment to more benign years^37^ may be driven by inter-annual variation in the strength and direction of winter storms (Fig. S1). What this may mean for restoring *P. australis* in these environments is that seeding may be an appropriate strategy for regenerating this species, but annual re-seeding over potentially a decade is recommended to capture inter-annual variation in both summer bottlenecks associated with bioturbators and grazers and winter bottlenecks from physical disturbances associated with winter storms (e.g.^9^).

We modelled the effect of seeding density on the number of surviving recruits across the entire study area and demonstrated that the probability of surviving from 100 seeds m^−2^ would be ~0.001. Although the probability of successful recruitment appears low and clearly below detectable limits for this seeding rate, it is comparable to recruitment success for terrestrial restoration species across a range of habitats at lower and higher seeding rates (Table 3). Simulating seeding densities 2- to 40-fold (Fig. 5a) resulted in a proportional increase in seedling establishment for *P. australis*, but assumes an unlimited carrying capacity. While increasing seeding densities may improve our power to detect recruitment success, seeding densities of 200–1 000 m^−2^ equate to what would be suitable based on seed size of *Posidonia* (2 cm). For other seagrass species with much smaller seeds, there have been contrasting effects of seeding density. For example^38^, found no consistent effects of seeding density on germination or emergence of *Z. marina* in field studies when seeded at rates as high as 1 250 seeds m^−2^, whereas controlled laboratory studies on *Cymodocea nodosa* found negative density dependence at much lower seeding rates, 44–666 seeds m^−2^ ^39^. Under field conditions, aggregations of established *Posidonia* seedlings have been found at densities of 10’s–100’s of seedlings per 0.1–0.2 m^−2^ within naturally formed pits and troughs in the sediment. These field observations suggest that *Posidonia* may benefit from higher seeding densities under specific micro-site conditions.

**Table 3:** Probability of seedling establishment within different habitat types.

Species	Habitat	Seeding rate (m^−2^)	Probability of seedling establishment	Authors
*Posidonia australis*	Marine (seagrass) Cockburn Sound, Perth Australia	100	0.001	This study
*Zostera marina*	Marine (seagrass) Chesapeake Bay, Virginia, USA	25–50	0.016–0.056	9
*Agropyron desertorum Elymus elymoides Pseudoroegenaria spicata*	Arid grasslands Eastern Oregon, USA	376	0–0.06	10
*Olea europaea*	Dense scrubland Sierra Sur de Jaén (Spain)	~37	0.002–0.009	51
*Rhamnus ludovici-salvatoris*	Scrubland Balearic Islands (western Mediterranean)	—	0.005–0.03	52
*Epilobium angustifolium Anaphalis margaritacea*,	Pumice Plains, Mount St. Helens Washington, USA	~285	0.014	53
*Vaccinium myrtillus*	Coniferous forest Southeast Sweden	1 250	0.003	37

### Conclusions and management implications

Evaluating life cycle processes are the first steps towards optimizing seagrass restoration programs. Targeted monitoring after seeding is needed to observe these early life-history stages and transitions, and will be particularly important when alternative management strategies are applied that aim to enhance vital rates that have a bearing on subsequent seedling survival^16,40^. We conclude that seedling recruitment in *Posidonia* populations is affected by site environmental conditions and frequency of disturbances prevailing within one month after seeding and some 4–6 months later when established seedlings are physically removed by winter storms. The differences observed across the environmental gradients demonstrate that patterns of plant recruitment can vary notably at a spatial and temporal scale, and that the constant pressures of disturbance across these scales limit population growth and restoration outcomes. Where these disturbances are moderated sufficiently or can be overcome through intervention strategies, seed-based restoration could replace other more labour intensive re-planting methods for seagrasses in general.

When utilizing seed as a restoration tool, this study showed we can use demographic models effectively to identify how and when ecological processes could be managed in order to alter seagrass population dynamics. If benthic fauna and winter storms are major drivers of seedling survival, then there are potential management options that can be used to overcome these limitations. For example, removal, relocation or deflection of the most problematic benthic fauna in wave-sheltered restoration sites during the early stages of seedling establishment could be used to reduce the probability of biotic dislodgement or predation during this vulnerable and critical life-stage (Johnson *et al*. unpublished data). Alternatively, increasing the initial seeding density could offset the early heavy losses through an ‘escape by predator satiation’ strategy, though this has largely been untested in seagrasses and relies upon greater investment of valuable seed resources. In wave-exposed locations, introducing seed and seedling mixtures of species whose niche habitats overlap with turbulent and high exposure environments could be a more effective approach to re-establish seagrass back into such environments. While the specific bioturbating fauna and threshold water velocities inhibiting establishment still need to be identified, our results suggest advances in these areas are likely to substantially increase our ability to restore seagrasses across a range of environments.

The temporal resolution (1–3 months) and spatial scale (144 km^2^) in a highly wave impacted environment over which this study was undertaken has provided greater perspective on the importance of environmental conditions suitable for *P. australis* seedling establishment. Of greater interest to seagrass restoration ecologists and practitioners is an ability to predict or forecast persistence within a set of environmental conditions. Projection models based on measured demographic rates is a valuable predictor of appropriate locations for seed introductions. By simulating several potential restoration-scale starting seed densities across our study area, outcomes from projection models suggest that successful establishment of seedlings would be restricted to the most favourable of environmental scenarios tested in this study; low energy sites. Low exposure habitats support the later stages of seedling development, although there still remains a potential bottleneck incurred soon after seeding as a result of predation by fauna. Notwithstanding, those seedlings that successfully navigate these filters during early life-stages tend to show long-term persistence, and eventually ramify across the seafloor within two years of seeding. Under moderate exposure scenarios, heavy losses incurred early in the establishment process have a disproportionate influence on seedling establishment and thus increasing initial seeding density will have little effect. However, we have less certainty on what is driving these early losses in these locations. Although we recorded high initial seedling survival in high exposure sites, recruitment was constrained by winter storms which impose a significant barrier to middle life stages of seedlings transitioning to established plants.

## Methodology

### Life stages and study location

To address these objectives we examined the variation in survival, impacts of predation and disturbance of seeds by animals and physical dislodgement from storms using a large experiment that direct-seeded *Posidonia australis* seeds across an environmental gradient within and outside Cockburn Sound, Western Australia. *Posidonia australis* is a large, habitat-forming seagrass that is one of the dominate features of nearshore coastal sediments across temperate and subtropical Australia^41^. This species produces large (15–20 mm) seeds that lack any seed dormancy (ie. direct-development) (summarized in^13^). After seed settlement the seed and seedlings undergo distinct early life-history stages and transitions prior to establishment as an adult plant (Fig. 1).

The study area, Cockburn Sound, is a marine embayment (16 km long and 9 km wide, 144 km^2^) located on the temperate west coast of Western Australia (Fig. 2). Cockburn Sound consists of a deep central basin (17–22 m deep) surrounded by shallow (1–15 m deep) platforms (south, east and west), and banks to the north (Parmelia and Success Banks). Seagrass meadows typically inhabit these platforms and banks and have been mapped accurately to a depth of 5 m^35,42^. There were substantial losses of seagrass on these platforms and banks during the 1960’s and 70’s due to eutrophication and industrial development of the coast, with minimal natural recovery within Cockburn Sound^42^ but significant recovery across Success and Parmelia Banks to the north^35^. Cockburn Sound is further characterised by a spatial and temporal gradient in wind-wave energy. Local water circulation within the Sound is driven by south-west winds during summer (sea breeze) and northerly and westerly storms during winter^34^. The southern end of the Sound is protected from south-westerly swells by Garden Island and Carnac Island. Northern locations on Parmelia Bank and Carnac Island are not protected from northerly and westerly swells (summer and winter). Study locations and specific site characteristics are described in Table S1, Supporting Information.

### Experimental design and measurements

In November 2013, approximately 22 000 *Posidonia australis* fruit were hand-collected from Woodman Point, Cockburn Sound. This location supports highly fecund and genetically diverse meadows of *P. australis* ^36^. This location represents a source of genetic material with proven dispersal capability to locations across the whole embayment and to the north^36,43^. Whole fruit were transported back to the lab where they were transferred into large (1800 L), temperature controlled (25 °C) recirculating aquaria. Vigorous aeration was applied to the aquaria to agitate floating fruit and promote fruit dehiscence and seed release^25^. Negatively buoyant seeds sink and were collected from the aquarium floor. Seedling viability was determined by the presence of an undamaged seed, intact prophyll and radical.

To determine how major abiotic and biotic characteristics, such as physical disturbance, depth and biotic interactions can potentially influence seed survival and recruitment we chose eight study locations with a gradient in wave exposure and depth (Table S1). Within each location three sites were selected. At each site, nine 1 m^2^ plots were established within a 5 m × 5 m area and plots were spaced 1 m apart. Three plots were randomly assigned full cage enclosures, three plots half-cage enclosures (procedural control) and three plots were uncaged controls. We direct-seeded 100 seedlings into each 1 m^2^ plot by gently inserting a developing seedling just below the sediment surface. This mimicked how seeds are buried naturally. Seeds were spaced equally in each plot. In total, 21 600 seedlings were planted into 216 m^2^ across an area encompassing 144 km^2^.

Seedling survival was monitored for 18 months at intervals that coincided with each life-stage transition. Life stages and transitions (Fig. 1) were defined by a changing dependency of the seedling on seed reserves during the first months of development^25,44,45^. Fruits mature and release an individual seed with an emerging radical and prophyll during late November. Subsequently, seedlings were planted in early December. Initially, seedlings are highly dependent on seed reserves; seed-dependent, Sd (December_yr1_ - January_yr1_). Seedlings then undergo an extended period where they continue to draw nourishment from maternally-derived reserves, however, during this stage there is greater uptake and assimilation of resources from the environment due to production of photosynthetically active leaves and development of a small but functional root system; autonomous development, Ad (January_yr1_–April_yr1_). By the end of this period seedlings have exhausted the majority (~90%) of their seed reserves and are relatively independent of their seed. Seedlings then become fully integrated into their environment upon exhaustion of the seed reserves; seedling establishment, Es (April_yr1_–September_yr1_). Production of new shoots, Ns (September_yr1_–year 2) typically occurs in the months following seedling establishment and seedlings become Juveniles. Juveniles transition into adults after plants undergo horizontal vegetative expansion, Ve (year 2–3). Adults typically become reproductively mature, f (fecundity), between years 3–5^7^.

During the experiment we monitored several biotic and abiotic characteristics at each location. A census on benthic faunal abundance was undertaken in February 2014, counting the number of animals present within a 5 m × 1 m transect, replicated six times at each site. The physical environment was also characterized (Table 1). Wave conditions were determined from pressure sensors (RBR Virtuoso, Dtide) deployed simultaneously over winter at different locations. Each sensor recorded pressure continuously at 1 Hz. Posterior analysis consisted of removing atmospheric pressure and converting recorded pressure to meters of water, we then separated the data into hourly bursts. A time series of water level was calculated and the trend removed to account only for events from the desired frequencies. A spectral density analysis for each burst was then calculated, focusing in the energy bands of the swell components with periods from 5 to 25 s (e.g.^46,47^). The significant wave height, *Hs*, defined as the highest third of the waves was calculated from the total spectral energy for the bursts across the mentioned frequencies. Sediment grain size distribution and sediment organic matter content (see Table S1) were determined from 9 cm diameter by 5 cm deep cores. Sediment cores were dried at 60 °C for 72 hours before compositional analysis. Sediment grain size distribution was analysed using settling velocities in a 2.2 m settling tube. The recorded settling velocity was transformed to sediment size using the Gibbs equation with the corresponding sand density of 2.56 g cm^−3^ measured by volumetric displacement. Sediment distribution was computed with the graphical logarithmic method from^48^. Sediment organic matter content was determined from loss on ignition at 550 °C following methods by^49^. Light (Photosynthetically Active Radiation, PAR) loggers (HOBO H21-002 Micro Station, recording every 5 minutes) and temperature loggers recording every minute (HOBO® UTBI-001 TidbiT, recording every 5 minutes) were deployed across the depth gradient; Southern Flats as the shallow site, Owen Anchorage was mid-depth and Parmelia Bank was the deep site (see Table S1).

### Statistical analysis

Mortality of early life-history transitions and how they vary spatially along gradients in disturbance were tested for normality. For transition probabilities, each 1 m^2^ plot was considered a replicate with Location (8), caging Treatment (3) and Life-Stage (4) as fixed factors and Site (3) nested within Location. A Bartletts test revealed the data were not normally distributed (R Core Team, 2014), so we used a fixed factor PERMANOVA (‘vegan’, R package) to test for differences in transition probabilities. Data were transformed into a Euclidean resemblance matrix, with 9999 permutations. If a significant main effect or interaction was detected we tested additional hypotheses about the pairwise differences in the coefficients. Pairwise comparisons revealed that cages confounded seedling survival across all Locations with the only exception being Owen Anchorage, where full caging treatments resulted in a positive effect on seedling survival for the first life stage. Subsequently, we simplified the model by removing the factor Treatment and re-analyzed the data using only control plots (ie. no cages) with Location and Life-Stage as fixed factors and Site nested within Location.

We used population matrices for each location to determine the influence of life stage on the overall recruitment dynamics. Because complete mortality occurred in some locations resulting in a zero probability of transitioning from one life-stage to the next, we replaced these values with a value of 10^−3^ which was lower than the probability of a seedling surviving within 1 m^2^ plot and had little weight on population growth. Although we only have values for the first five stages of this matrix, we populated the sixth transition or mortality and the fecundity (R1, Fig. 1) from data collected at Rottnest Island on seed production and natural seedling mortality over multiple years (Kendrick unpublished). A value of 0.2 was assigned as mortality and remained constant across all models, and we used a value of 8 as a fecundity value based on seed production (Kendrick unpublished). While these mortality and fecundity values may not represent the sites tested in this study, we tested the conclusions drawn from our matrix and sensitivity analysis by varying adult transition rates within realistic values; fecundity from 3 to 15 and then mortality from 0.06 to 0.5 (Supplementary Information Tables S2–S18). Despite modifying fecundity and mortality within realistic values our conclusions regarding the early life-stages as bottlenecks to seed-based restoration success do not change. However, only when adult mortality reaches a threshold of 0.5 or greater (mortality of half the adult population each year) does the adult life-stage become a bottleneck. We then calculated the population growth rate (λ) as the dominant eigenvalue of each transition matrix and then calculated the sensitivity and elasticity of each transition using Matlab code “eigenall”. These values determine the overall importance of individual transitions to λ based on the left and right eigenvectors^50^. We finally modelled the transition matrices to determine what the outcome would have been if we increased starting seed densities across the gradient in environmental conditions and observed the outcome of seed survival. We modelled starting seed densities of 100 m^2^, 200 m^2^, 500 m^2^, 1000 m^2^ and 10,000 m^2^.

## Electronic supplementary material

Supplementary Information
