# Supplementary material for: Identifying critical recruitment bottlenecks limiting seedling establishment in a degraded seagrass ecosystem
Source: Sci Rep. 2017 Nov 1;7:14786. doi: 10.1038/s41598-017-13833-y (PMC5665928; doi:10.1038/s41598-017-13833-y)
Supplement: Supplementary file 1 — Supplementary Information [file 41598_2017_13833_MOESM1_ESM.pdf]

Identifying critical recruitment bottlenecks limiting seedling establishment in a degraded seagrass ecosystem

*John Statton[1] - john.statton@uwa.edu.au (Mob) ++61 4 23 393 677 (W) ++61 8 6488 2306

Leonardo R. Montoya[1] - leonardo.ruizmontoya@uwa.edu.au

Robert J. Orth[2] - jjorth@vims.edu

Kingsley W. Dixon[3] - kingsley.dixon@curtin.edu.au

Gary A. Kendrick[1] - gary.kendrick@uwa.edu.au

[1]School of Biological Sciences and UWA Oceans Institute, Faculty of Natural and Agricultural Science, University of Western Australia, Crawley, 6009, Perth, Western Australia

[2]Virginia Institute of Marine Science, College of William and Mary, Gloucester Pt., 23061, VA, USA

[3]Department of Environment and Agriculture, Curtin University, Bentley, 6102, Perth, Western Australia

*Correspondence to John Statton (john.statton@uwa.edu.au)

Running title: Recruitment bottlenecks in seagrass seedlings

Table S1: Environmental characteristics (water depth, significant wave height, sediment grain size and organic matter content, benthic light availability and temperature) at selected locations (Southern Flats (SF), southern flats edge (SFe), Cockburn Sound east bank (CS), Woodman Point (WP), Owen Anchorage (OA), Garden Island (GI), Carnac Island (CI), and Parmelia Bank (PB)). Values are means (±1 SE).

Location	Water depth (m)	Significant wave height (m)	Sediment grain-size (mm)	Sediment organic matter (% DW)	Daily irradiance (PAR, mols photons m^{-2} d^{-1})	Temperature (^{o}C)
SF	2.41	0.08	0.4±0.02	2.27±0.15	10.69±1.96	
SFe	2.72					
CS	7.48	0.14	0.31±0.01	2.8±0.26		22.90±0.60
WP	4.29	0.24	0.30±0.03	2.5±0.10		
OA	4.55	0.19	0.36±0.00	2.73±0.32	7.10±1.72	22.56±0.25
GI	2.52		0.23±0.07	3.03±0.12		
CI	2.95	0.31	0.27±0.04	2.93±0.15		
PB	8.42	0.46	0.27±0.01	2.6±0.36	3.28±0.91	22.27±0.27

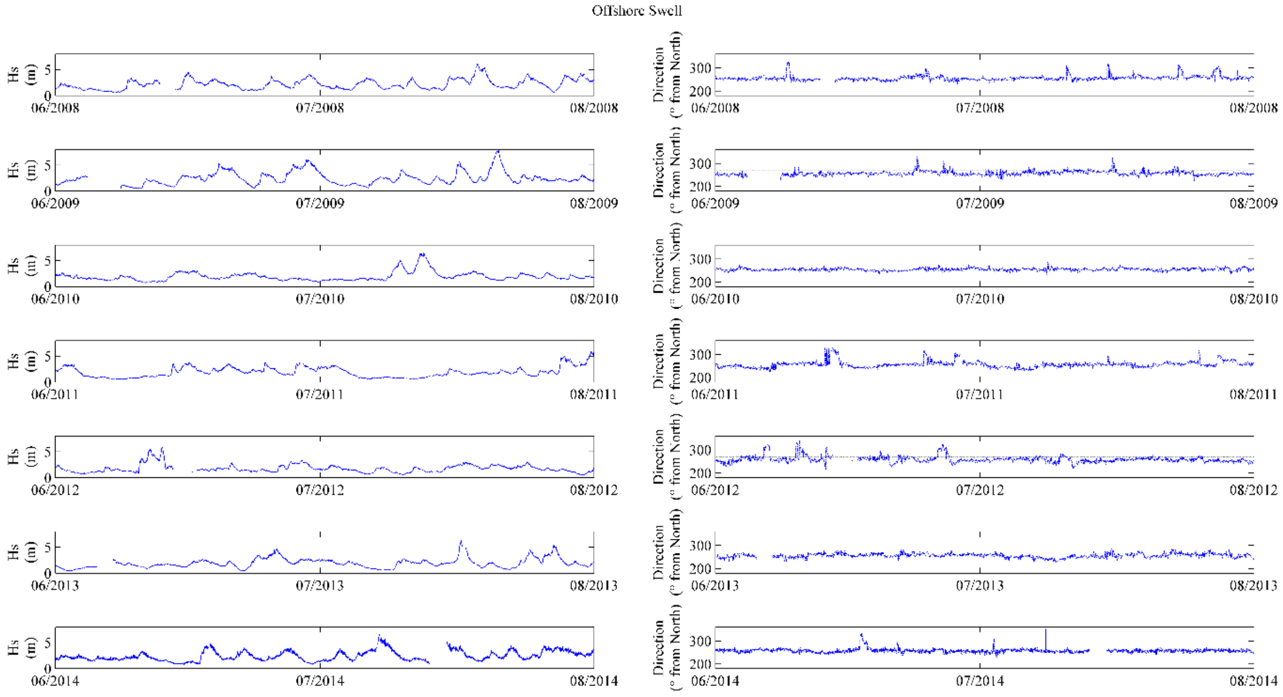

Figure S1: Historical significant wave height (Hs) and direction for the swell components at Parmelia Bank, Western Australia. (left) Each major peak represents a large storm system

(arriving from any direction) from 2008 – 2014; (right) each major peak represents a large storm system arriving from the north.

Table S2 – S18: S2 (original data supplied in the publication for reference); S3 – S7 (adult fecundity values altered); S8 – S14 (adult mortality values altered); S15 – S18 (adult fecundity and mortality values altered). Highlighted cells: Blue = the life stages with the greatest sensitivities; Red = when the vegetative elasticity is lower than other life stages; Green = the vegetative elasticity is greater than other life stages; Yellow = the threshold of change (ie. when a change in value for adult mortality and/or fecundity causes a greater change than the original values used in our statistical analysis for publication.

Table S2

ORIGINAL FOR PUBLICATION mortality = 0.2 fecundity = 8

		Sensitivities & Elasticities						
	λ		Sd	Ad	Es	Ns	Ve	f
All sites as a single population	**0.49**	*S=*	***0.62***	0.23	***2.17***	0.09	0.3	0.01
		E=	0.18	0.18	0.18	0.18	0.12	0.18
Low exposure; Shallow (SF, SFe)	0.21	*S=*	***8.24***	*2.29*	0	0	0.91	0
		E=	0.02	0.02	0.02	0.02	0.89	0.02
Low exposure; Deep (CS)	**0.71**	*S=*	***1.37***	*0.68*	0.15	0.13	0.26	0.02
		E=	0.19	0.19	0.19	0.19	0.07	0.19
Moderate exposure and depth (WP & OA)	0.21	*S=*	***4.12***	***4.58***	0	0	0.91	0
		E=	0.02	0.02	0.02	0.02	0.89	0.02
High exposure; Shallow (GI,CI)	0.21	*S=*	0.01	0.01	**538.22**	0	*0.91*	0
		E=	0.02	0.02	0.02	0.02	0.89	0.02
High exposure; Deep (PB)	0.21	*S=*	0.02	0.01	**581.74**	0	*0.91*	0
		E=	0.02	0.02	0.02	0.02	0.89	0.02

Table S3

	fecundity = 3					
λ	Sd	Ad	Es	Ns	Ve	f
**0.41**	***0.50***	0.18	***1.76***	0.07	0.33	0.02
	0.17	0.17	0.17	0.17	0.16	0.17
0.20	***3.46***	*0.96*	0.00	0.00	0.96	0.00
	0.01	0.01	0.01	0.01	0.95	0.01
**0.60**	***1.12***	*0.55*	0.12	0.11	0.27	0.04
	0.18	0.18	0.18	0.18	0.09	0.18
0.20	***1.73***	***1.93***	0.00	0.00	0.96	0.00
	0.01	0.01	0.01	0.01	0.95	0.01
0.20	0.01	0.01	**226.26**	0.00	*0.96*	0.00

	0.01	0.01	0.01	0.01	0.95	0.01
0.20	0.01	0.00	**244.55**	0.00	*0.96*	0.00
	0.01	0.01	0.01	0.01	0.95	0.01

Table S4

	fecundity = 5					
λ	Sd	Ad	Es	Ns	Ve	f
**0.45**	***0.56***	0.20	***1.97***	0.08	0.31	0.02
	0.17	0.17	0.17	0.17	0.14	0.17
0.20	***5.50***	*1.53*	0.00	0.00	0.94	0.00
	0.02	0.02	0.02	0.02	0.92	0.02
**0.65**	***1.24***	*0.62*	0.14	0.12	0.26	0.02
	0.18	0.18	0.18	0.18	0.08	0.18
0.20	***2.75***	***3.06***	0.00	0.00	0.94	0.00
	0.02	0.02	0.02	0.02	0.92	0.02
0.20	0.01	0.01	**359.45**	0.00	*0.94*	0.00
	0.02	0.02	0.02	0.02	0.92	0.02
0.20	0.01	0.01	**388.51**	0.00	*0.94*	0.00
	0.02	0.02	0.02	0.02	0.92	0.02

Table S5

	fecundity = 7					
λ	Sd	Ad	Es	Ns	Ve	f
**0.48**	***0.61***	0.22	***2.11***	0.08	0.30	0.01
	0.17	0.17	0.17	0.17	0.13	0.17
0.20	***7.36***	*2.05*	0.00	0.00	0.92	0.00
	0.02	0.02	0.02	0.02	0.90	0.02
**0.70**	***1.33***	*0.66*	0.15	0.13	0.26	0.02
	0.19	0.19	0.19	0.19	0.07	0.19
0.20	***3.68***	***4.10***	0.00	0.00	0.92	0.00
	0.02	0.02	0.02	0.02	0.90	0.02
0.20	0.01	0.01	**481.14**	0.00	*0.92*	0.00
	0.02	0.02	0.02	0.02	0.90	0.02
0.20	0.02	0.01	**520.04**	0.00	*0.92*	0.00
	0.02	0.02	0.02	0.02	0.90	0.02

Table S6

	fecundity = 11					
λ	Sd	Ad	Es	Ns	Ve	f

**0.52**	***0.67***	0.24	***2.33***	0.09	0.29	0.01
	0.18	0.18	0.18	0.18	0.11	0.18
0.21	***10.66***	*2.97*	0.01	0.01	0.89	0.00
	0.03	0.03	0.03	0.03	0.86	0.03
**0.76**	***1.46***	*0.72*	0.16	0.14	0.25	0.01
	0.19	0.19	0.19	0.19	0.07	0.19
0.21	***5.33***	***5.93***	0.01	0.01	0.89	0.00
	0.03	0.03	0.03	0.03	0.86	0.03
0.21	0.02	0.02	**696.53**	0.01	*0.89*	0.00
	0.03	0.03	0.03	0.03	0.86	0.03
0.21	0.02	0.01	**752.85**	0.01	*0.89*	0.00
	0.03	0.03	0.03	0.03	0.86	0.03

Table S7

	fecundity = 15					
λ	Sd	Ad	Es	Ns	Ve	f
**0.55**	***0.71***	0.26	***2.48***	0.10	0.28	0.01
	0.18	0.18	0.18	0.18	0.10	0.18
0.21	***13.51***	*3.76*	0.01	0.01	0.86	0.00
	0.04	0.04	0.04	0.04	0.82	0.04
**0.80**	***1.56***	*0.77*	0.17	0.15	0.25	0.01
	0.19	0.19	0.19	0.19	0.06	0.19
0.21	***6.76***	***7.51***	0.01	0.01	0.86	0.00
	0.04	0.04	0.04	0.04	0.82	0.04
0.21	0.02	0.02	**882.50**	0.01	*0.86*	0.00
	0.04	0.04	0.04	0.04	0.82	0.04
0.21	0.03	0.01	**953.85**	0.01	*0.86*	0.00
	0.04	0.04	0.04	0.04	0.82	0.04

Table S8

	mortality = 0.05					
λ	Sd	Ad	Es	Ns	Ve	f
**0.45**	***0.64***	0.23	***2.23***	0.09	0.22	0.01
	0.20	0.20	0.20	0.20	0.02	0.20
0.11	***34.00***	*9.46*	0.02	0.02	0.31	0.00
	0.17	0.17	0.17	0.17	0.14	0.17
**0.68**	***1.38***	*0.68*	0.15	0.13	0.21	0.02
	0.20	0.20	0.20	0.20	0.02	0.20
0.11	***17.01***	***18.91***	0.02	0.02	0.31	0.00

	0.17	0.17	0.17	0.17	0.14	0.17
0.11	0.05	0.05	**2221.38**	0.02	*0.31*	0.00
	0.17	0.17	0.17	0.17	0.14	0.17
0.11	0.07	0.03	**2400.98**	0.02	*0.31*	0.00
	0.17	0.17	0.17	0.17	0.14	0.17

Table S9

	mortality = 0.06					
λ	Sd	Ad	Es	Ns	Ve	f
**0.45**	***0.64***	0.23	***2.22***	0.09	0.22	0.01
	0.19	0.19	0.19	0.19	0.03	0.19
0.11	***33.35***	*9.28*	0.02	0.02	0.35	0.00
	0.16	0.16	0.16	0.16	0.18	0.16
**0.68**	***1.38***	*0.68*	0.15	0.13	0.22	0.02
	0.20	0.20	0.20	0.20	0.02	0.20
0.11	***16.68***	***18.56***	0.02	0.02	0.35	0.00
	0.16	0.16	0.16	0.16	0.18	0.16
0.11	0.05	0.05	**2179.17**	0.02	*0.35*	0.00
	0.16	0.16	0.16	0.16	0.18	0.16
0.11	0.07	0.03	**2355.36**	0.02	*0.35*	0.00
	0.16	0.16	0.16	0.16	0.18	0.16

Table S10

	mortality = 0.1					
λ	Sd	Ad	Es	Ns	Ve	f
**0.46**	***0.63***	0.23	***2.22***	0.09	0.24	0.01
	0.19	0.19	0.19	0.19	0.05	0.19
0.13	***28.39***	*7.90*	0.02	0.02	0.52	0.00
	0.12	0.12	0.12	0.12	0.40	0.12
**0.69**	***1.38***	*0.68*	0.15	0.13	0.23	0.02
	0.19	0.19	0.19	0.19	0.03	0.19
0.13	***14.20***	***15.80***	0.02	0.02	0.52	0.00
	0.12	0.12	0.12	0.12	0.40	0.12
0.13	0.04	0.05	**1855.12**	0.02	*0.52*	0.00
	0.12	0.12	0.12	0.12	0.40	0.12
0.13	0.06	0.03	**2005.11**	0.02	*0.52*	0.00
	0.12	0.12	0.12	0.12	0.40	0.12

Table S11

	mortality = 0.15					
**0.48**	***0.63***	0.23	***2.20***	0.09	0.27	0.01
	0.18	0.18	0.18	0.18	0.08	0.18
0.16	***17.39***	*4.84*	0.01	0.01	0.76	0.00
	0.06	0.06	0.06	0.06	0.70	0.06
**0.70**	***1.38***	*0.68*	0.15	0.13	0.24	0.02
	0.19	0.19	0.19	0.19	0.05	0.19
0.16	***8.70***	***9.68***	0.01	0.01	0.76	0.00
	0.06	0.06	0.06	0.06	0.70	0.06
0.16	0.03	0.03	**1136.40**	0.01	*0.76*	0.00
	0.06	0.06	0.06	0.06	0.70	0.06
0.16	0.04	0.02	**1228.28**	0.01	*0.76*	0.00
	0.06	0.06	0.06	0.06	0.70	0.06

Table S12

	mortality = 0.25					
λ	Sd	Ad	Es	Ns	Ve	f
**0.51**	***0.61***	0.22	***2.14***	0.08	0.33	0.01
	0.17	0.17	0.17	0.17	0.16	0.17
0.25	***3.82***	*1.06*	0.00	0.00	0.97	0.00
	0.01	0.01	0.01	0.01	0.96	0.01
**0.73**	***1.36***	*0.67*	0.15	0.13	0.28	0.02
	0.18	0.18	0.18	0.18	0.09	0.18
0.25	***1.91***	***2.12***	0.00	0.00	0.97	0.00
	0.01	0.01	0.01	0.01	0.96	0.01
0.25	0.01	0.01	**249.49**	0.00	*0.97*	0.00
	0.01	0.01	0.01	0.01	0.96	0.01
0.25	0.01	0.00	**269.66**	0.00	*0.97*	0.00
	0.01	0.01	0.01	0.01	0.96	0.01

Table S13

	mortality = 0.3					
λ	Sd	Ad	Es	Ns	Ve	f
**0.52**	***0.60***	0.22	***2.08***	0.08	0.37	0.01
	0.16	0.16	0.16	0.16	0.21	0.16
0.30	***1.92***	*0.53*	0.00	0.00	0.99	0.00

	0.00	0.00	0.00	0.00	0.98	0.00
**0.74**	***1.35***	*0.67*	0.15	0.13	0.30	0.02
	0.18	0.18	0.18	0.18	0.12	0.18
0.30	***0.96***	***1.07***	0.00	0.00	0.99	0.00
	0.00	0.00	0.00	0.00	0.98	0.00
0.30	0.00	0.00	**125.30**	0.00	*0.99*	0.00
	0.00	0.00	0.00	0.00	0.98	0.00
0.30	0.00	0.00	**135.43**	0.00	*0.99*	0.00
	0.00	0.00	0.00	0.00	0.98	0.00

Table S14

	mortality = 0.5					
**0.62**	***0.48***	0.17	***1.67***	0.07	0.57	0.01
	0.11	0.11	0.11	0.11	0.46	0.11
0.50	***0.26***	*0.07*	0.00	0.00	1.00	0.00
	0.00	0.00	0.00	0.00	1.00	0.00
**0.81**	***1.27***	*0.63*	0.14	0.12	0.40	0.02
	0.15	0.15	0.15	0.15	0.24	0.15
0.50	***0.13***	***0.14***	0.00	0.00	1.00	0.00
	0.00	0.00	0.00	0.00	1.00	0.00
0.50	0.00	0.00	**16.67**	0.00	*1.00*	0.00
	0.00	0.00	0.00	0.00	1.00	0.00
0.50	0.00	0.00	**18.02**	0.00	*1.00*	0.00
	0.00	0.00	0.00	0.00	1.00	0.00

Table S15

	mortality = 0.1 fecundity = 4					
λ	Sd	Ad	Es	Ns	Ve	f
**0.41**	***0.55***	0.20	***1.93***	0.08	0.25	0.02
	0.19	0.19	0.19	0.19	0.06	0.19
0.12	***22.18***	*6.17*	0.01	0.01	0.59	0.00
	0.10	0.10	0.10	0.10	0.49	0.10
**0.60**	***1.20***	*0.59*	0.13	0.12	0.23	0.03
	0.19	0.19	0.19	0.19	0.04	0.19
0.12	***11.09***	***12.34***	0.01	0.01	0.59	0.00
	0.10	0.10	0.10	0.10	0.49	0.10
0.12	0.03	0.04	**1449.17**	0.01	*0.59*	0.00
	0.10	0.10	0.10	0.10	0.49	0.10

0.12	0.05	0.02	**1566.34**	0.01	*0.59*	0.00
	0.10	0.10	0.10	0.10	0.49	0.10

Table S16

	mortality = 0.1 fecundity = 12					
λ	Sd	Ad	Es	Ns	Ve	f
**0.50**	***0.69***	0.25	***2.41***	0.10	0.24	0.01
	0.19	0.19	0.19	0.19	0.05	0.19
0.14	***32.20***	*8.96*	0.02	0.02	0.48	0.00
	0.13	0.13	0.13	0.13	0.35	0.13
**0.75**	***1.50***	*0.74*	0.16	0.14	0.22	0.01
	0.19	0.19	0.19	0.19	0.03	0.19
0.14	***16.11***	***17.92***	0.02	0.02	0.48	0.00
	0.13	0.13	0.13	0.13	0.35	0.13
0.14	0.05	0.05	**2104.02**	0.02	*0.48*	0.00
	0.13	0.13	0.13	0.13	0.35	0.13
0.14	0.07	0.03	**2274.13**	0.02	*0.48*	0.00
	0.13	0.13	0.13	0.13	0.35	0.13

Table S17

	mortality = 0.27 fecundity = 4					
λ	Sd	Ad	Es	Ns	Ve	f
**0.46**	***0.52***	0.19	***1.80***	0.07	0.38	0.02
	0.16	0.16	0.16	0.16	0.22	0.16
0.27	***1.47***	*0.41*	0.00	0.00	0.99	0.00
	0.00	0.00	0.00	0.00	0.98	0.00
**0.65**	***1.17***	*0.58*	0.13	0.11	0.30	0.03
	0.18	0.18	0.18	0.18	0.12	0.18
0.27	***0.73***	***0.82***	0.00	0.00	0.99	0.00
	0.00	0.00	0.00	0.00	0.98	0.00
0.27	0.00	0.00	**95.90**	0.00	*0.99*	0.00
	0.00	0.00	0.00	0.00	0.98	0.00
0.27	0.00	0.00	**103.66**	0.00	*0.99*	0.00
	0.00	0.00	0.00	0.00	0.98	0.00

Table S18

	mortality = 0.27 fecundity = 12					
λ	Sd	Ad	Es	Ns	Ve	f
**0.55**	***0.66***	0.24	***2.32***	0.09	0.33	0.01

	0.17	0.17	0.17	0.17	0.16	0.17
0.27	***4.20***	*1.17*	0.00	0.00	0.97	0.00
	0.01	0.01	0.01	0.01	0.96	0.01
**0.79**	***1.48***	*0.73*	0.16	0.14	0.28	0.01
	0.18	0.18	0.18	0.18	0.09	0.18
0.27	***2.10***	***2.34***	0.00	0.00	0.97	0.00
	0.01	0.01	0.01	0.01	0.96	0.01
0.27	0.01	0.01	**274.69**	0.00	*0.97*	0.00
	0.01	0.01	0.01	0.01	0.96	0.01
0.27	0.01	0.00	**296.90**	0.00	*0.97*	0.00
	0.01	0.01	0.01	0.01	0.96	0.01
